# Interplay between ROS and Antioxidants during Ischemia-Reperfusion Injuries in Cardiac and Skeletal Muscle

**DOI:** 10.3390/ijms19020417

**Published:** 2018-01-31

**Authors:** Tingyang Zhou, Evan R. Prather, Davis E. Garrison, Li Zuo

**Affiliations:** 1 Radiologic Sciences and Respiratory Therapy Division, School of Health and Rehabilitation Sciences, The Ohio State University College of Medicine, The Ohio State University Wexner Medical Center, Columbus, OH 43210, USA; zhou.1273@osu.edu (T.Z.); prather.50@buckeyemail.osu.edu (E.R.P.); garrison.189@buckeyemail.osu.edu (D.E.G.); 2 Interdisciplinary Biophysics Graduate Program, The Ohio State University, Columbus, OH 43210, USA

**Keywords:** contraction, ischemic preconditioning, free radicals, cytochrome *c*

## Abstract

Ischemia reperfusion (IR), present in myocardial infarction or extremity injuries, is a major clinical issue and leads to substantial tissue damage. Molecular mechanisms underlying IR injury in striated muscles involve the production of reactive oxygen species (ROS). Excessive ROS accumulation results in cellular oxidative stress, mitochondrial dysfunction, and initiation of cell death by activation of the mitochondrial permeability transition pore. Elevated ROS levels can also decrease myofibrillar Ca^2+^ sensitivity, thereby compromising muscle contractile function. Low levels of ROS can act as signaling molecules involved in the protective pathways of ischemic preconditioning (IPC). By scavenging ROS, antioxidant therapies aim to prevent IR injuries with positive treatment outcomes. Novel therapies such as postconditioning and pharmacological interventions that target IPC pathways hold great potential in attenuating IR injuries. Factors such as aging and diabetes could have a significant impact on the severity of IR injuries. The current paper aims to provide a comprehensive review on the multifaceted roles of ROS in IR injuries, with a focus on cardiac and skeletal muscle, as well as recent advancement in ROS-related therapies.

## 1. Introduction

Ischemia reperfusion (IR) injury occurs when tissues or organs are subjected to a period of ischemia, followed by blood replenishment. It has become a common and critical clinical issue in myocardial infarction, organ transplants, and extremity injuries and causes damage to various organs such as the brain, heart, and skeletal muscles [1]. The molecular mechanisms underlying IR injury have been extensively investigated over the past decades. It has been evidenced that reactive oxygen species (ROS) as well as the downstream activated cytokines may play major roles in IR injury [1]. ROS are oxygen-containing reactive molecules that can be naturally generated from biological systems. When produced at low levels, ROS act as important signaling molecules involved in a variety of physiological activities such as immune response, muscle contraction, and exercise adaptation [2]. Endogenous antioxidants including catalase, superoxide dismutase (SOD), glutathione, and glutathione peroxidase (GPx) are important ROS scavengers that are responsible for maintaining ROS at normal levels. However, under specific pathological conditions, antioxidant defenses can be overwhelmed, resulting in cellular oxidative stress [2]. In addition, ROS may induce secondary ROS release via the activation of immune cells and the disruption of mitochondrial function. Elevated ROS levels may cause peroxidation of proteins, DNA, and lipids, and can trigger mitochondria-induced cell death pathways, thereby contributing to IR injury [2,3,4,5]. Despite these detrimental effects, evidence has indicated that related therapies for IR injury such as pre- and postconditioning rely heavily on the induction of ROS as signaling molecules to improve cell survival [6].

Although there has been an abundant amount of literature describing the roles of ROS in mediating IR injuries, the molecular mechanisms have not been fully elucidated in the context of cardiac and skeletal muscle. There are numerous similarities between the cardiac and skeletal muscle in IR-induced damage pathways due to their characteristic striation, but there are also differences. IR-induced injury in skeletal muscle is inevitable under numerous clinical conditions such as in musculoskeletal and vascular traumas, time-consuming transplantation, and musculoskeletal and vascular reconstructive surgeries [7,8]. Specifically, the “no-reflow” phenomenon is commonly observed in replantation of traumatic amputations, where blood flow is seriously impaired at microcirculation levels even after the releasing of artery occlusion. It was reported that “no-reflow” phenomenon accounts for 58% of the digital replantation failures [7]. Although skeletal muscle is relatively tolerant to ischemia as compared to other organs, prolonged ischemia can cause irreversible complications such as myocyte necrosis and apoptosis [7,8]. Therefore, early re-introduction of blood flow to the ischemic area is one of the primary treatment goals for tissue salvage. However, a burst of ROS are produced upon the initiation of reperfusion, which can cause IR injury beyond that induced by ischemia [7]. Further research is needed to understand the contributions of ROS in cardiac and skeletal myocytes to develop more efficient treatments. This article will provide an updated summary of the complicated roles of ROS in mediating IR injury, with a focus on cardiac and skeletal muscles. We will also discuss the novel protective approaches used to manage IR-induced oxidative stress and associated molecular mechanisms. The last section will review the physiopathology of IR in the elderly populations as well as patients with diabetes mellitus, as the severity of IR injury has been shown to be related with these two conditions [9,10].

## 2. Reactive Oxygen Species (ROS) in Cardiac and Skeletal Muscles

### 2.1. Roles of ROS in Cardiac Muscle

Under physiological conditions, the primary cellular sources of ROS in striated muscles include mitochondria, xanthine oxidase (XO), and NADPH oxidase (NOX) [2]. In the heart, ROS generation can also be stimulated in response to growth factors and cytokines such as angiotensin II (ATII), tumor necrosis factor-α, and transforming growth factors [11,12]. Abundant evidence has suggested that significant ROS production can be triggered by the exposure to stress stimuli or heart failure. Interestingly, antioxidant administration in experimental and clinical trials has yielded mixed and inconsistent results, suggesting the importance for further research in this area [11,13,14]. Cohort studies, however, have generally found a positive correlation of antioxidants like flavonoids and α and β-carotene with reduced cardiovascular disease (CVD)-related mortality [13]. Additionally, it has been reported that ATII can cause cardiac hypertrophy involving ROS and ROS-initiated mitogen-activated protein kinases (MAPKs) activation, whereas such hypertrophy can be attenuated by antioxidant treatment [11]. ROS have also been implicated in the regulation of muscle contractibility by altering ion flux in cardiomyocytes. Specifically, excessive ROS formation was found to decrease the activity of sarcoplasmic reticulum Ca^2+^ ATPase 2 (SERCA2) and reduce myofibrillar calcium (Ca^2+^) sensitivity [15,16]. As a result, prolonged exposure to ROS may compromise the excitation-contraction coupling in myocytes, thereby promoting the progression of heart failure [11].

### 2.2. Roles of ROS in Skeletal Muscle

While it was initially thought that mitochondria were the most significant contributors of ROS in skeletal muscle, recent data have suggested that NOX enzymes located on the sarcoplasmic reticulum (SR), plasma membrane, transverse tubules, and triads may play important roles in ROS production during muscle contraction [17,18]. Accordingly, Sakellariou et al. found that mitochondria do not contribute significantly to ROS production during short-term isometric contractions. Rather, NOX was found to be the major source of intracellular ROS production [19]. In contractile skeletal muscle, ROS production is markedly boosted due to the enhanced enzyme activity of NOX and XO [20]. Superoxide (O_2_^•−^) has been found to be one of the most abundant ROS produced in contracting skeletal muscle. In addition, O_2_^•−^ can act as precursors to produce secondary ROS such as hydrogen peroxide (H_2_O_2_) and hydroxyl radical [17].

Increased ROS released from the contractile skeletal muscles have been correlated with fatigue development. This is evidenced by the ability of antioxidants to delay muscle dysfunction [21]. For example, Tiron treatment can markedly attenuate skeletal muscle fatigue induced by repeated tetanic contraction at 37 °C in mice [22]. ROS have been shown to play a critical role in fatigue-induced force depression, particularly at low frequencies. It was shown in a rat model that increased myoplasmic ROS accumulated during tetanic stimulation in muscle fibers, which was accompanied by marked force decline [23]. The detrimental effects of ROS on skeletal muscle function could be attributed to their ability to decrease myofibril Ca^2+^ sensitivity [22]. This was supported by in vitro studies that demonstrated treatment of a single myocyte with H_2_O_2_ decreased Ca^2+^ sensitivity of the myofibril. This can be reversed by the administration of reducing agents (e.g., dithiothreitol) [24]. The resistance of skeletal muscle to fatigue can be compromised after IR treatment potentially due to excessive ROS formation [25]. Further research suggested that muscle fatigue resistance remained reduced 14 days after IR in mice and muscle function was still not fully recovered after 56 days [26]. This prolonged muscle functional decline could be associated with sustained oxidative stress and inflammatory response induced by IR [26]. Antioxidants such as *S*-nitroso-*N*-acetylcysteine were shown to effectively protect skeletal muscle contractile function following IR injuries [25].

Despite these detrimental effects, a small amount of ROS generation is critical for normal skeletal muscle contraction [18]. For instance, NOX-induced ROS are involved in the activation of Ca^2+^-mediated intracellular cascades, such as the extracellular signal-regulated kinases (ERK)1/2 and Jun amino-terminal kinases (JNK). The triggering of this pathway is responsible for exercise-induced adaptation [27,28]. Another important role of ROS in skeletal muscles involves the facilitation of glucose transport into myocytes during exercise [29]. It was found that glucose uptake was enhanced threefold during muscle contraction. However, the improved glucose transport efficiency was significantly suppressed by *N*-acetylcysteine (NAC, a type of antioxidant) treatment. It has been suggested that ROS may play a key role in the contraction-induced glucose transport mechanism via the activation of AMP-activated protein kinase (AMPK) [29]. These dual roles of ROS in regulating muscle function may be dependent on the concentrations of ROS and specific physiological conditions.

## 3. ROS-Mediated Damage during IR

Skeletal muscle is much more tolerant to ischemia than the myocardium, as severe injury appears after 3 h of ischemia in skeletal muscle while only 20–40 min of ischemia causes irreversible damage to the myocardium [30]. A major source of mitochondrial damage in skeletal muscle during ischemia is due to the metabolic and ionic changes caused by a lack of oxygen in the blood. This leads to a reduction in oxidative phosphorylation and a decrease in ATP synthesis [30]. Low ATP concentrations then lead to dysfunction in ionic exchangers (Na^+^/K^+^-ATPases and Ca^2+^-ATPases) and reversal of Na^+^/Ca^2+^ antiporter mechanism, thereby resulting in an accumulation of cytosolic Ca^2+^. These elevated intracellular Ca^2+^ levels are responsible for irreversible damage to cell integrity due to the activation of cellular degradation enzymes such as lysozymes and phospholipases [30].

Increasing evidence has shown that ROS accumulate rapidly at the beginning of ischemia in striated muscle despite limited O_2_ supply, during which XO is a major contributor to ROS production [6,31]. As shown in reaction (1), in the presence of XO and oxygen (O_2_), hypoxanthine can be converted to xanthine, which simultaneously produces O_2_^•−^ [30]. Since XO and hypoxanthine are quickly accumulated during ischemia, this results in increased O_2_^•−^ production. It is interesting to note that during ischemia, molecular O_2_ is the limited substrate for this reaction but can be available upon reperfusion [1]. This could justify greater ROS formation during reperfusion than during ischemia [31,32]. Additionally, upon reperfusion, a process of reoxygenation, damaged mitochondria produce excessive ROS, resulting in the elevation of cytosolic Ca^2+^ concentration as well as cell death [30]. Furthermore, it has been indicated that myoglobin autoxidation that occurs in response to elevated NADH during ischemia is another potential source of ROS in cardiac muscles [31,33].

(1) $$\mathrm{Hypoxanthine}+O_{2}\overset{\mathrm{XO}}{\to }\mathrm{Xanthine}+\;O_{2}^{.-}$$

The sudden influx of oxygen during reperfusion is the primary cause of myocyte death, as it results in excessive quantities of various ROS with reduced antioxidant defense due to ischemia. Reperfusion also triggers the opening of mitochondrial permeability transition pores (mPTP), which results in cell death due to mitochondrial swelling and cell rupture. Additionally, ROS produced during reperfusion trigger an intense proinflammatory response that causes further muscle damage. The recruitment of neutrophils during inflammation produces additional ROS through NOX enzymes and leads to lipid peroxidation [30].

### 3.1. ROS-Mediated IR Injury in Cardiac Muscle

**Figure 1:**
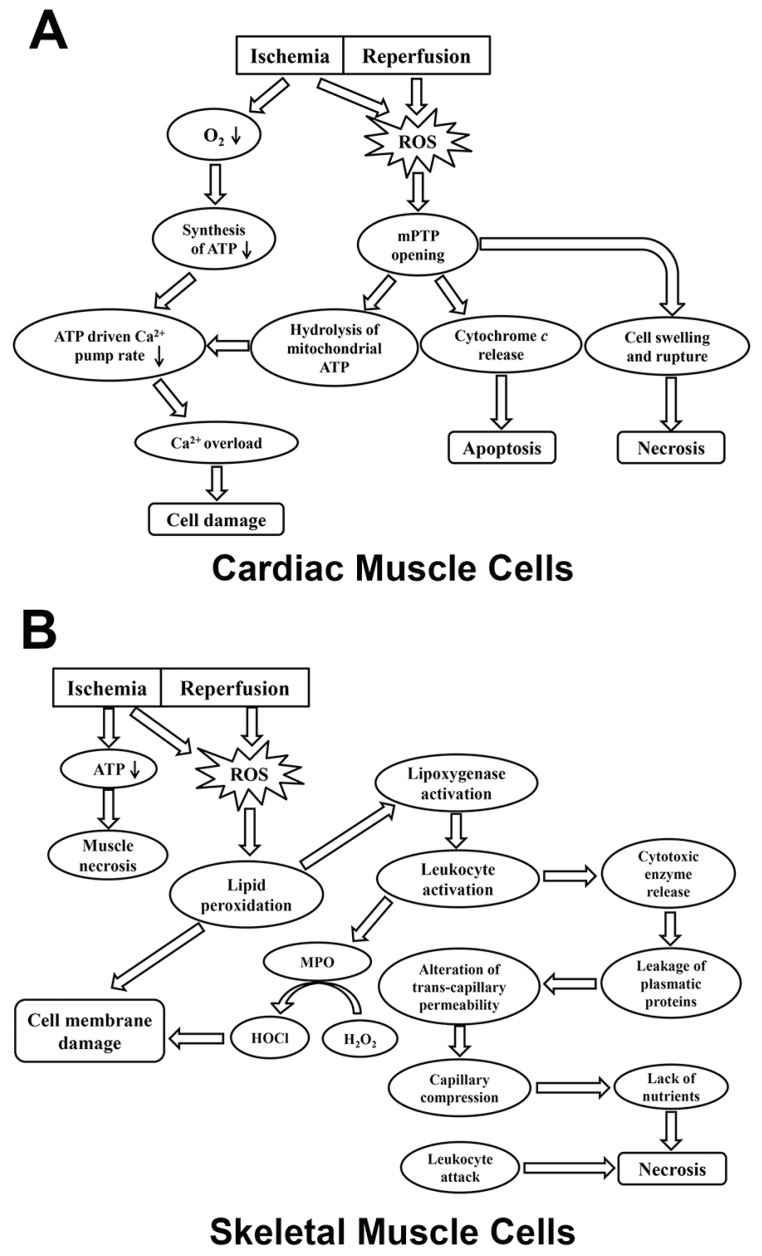
Schematic illustrating ROS-involved mechanisms underlying IR injury in cardiac muscle cells (**A**) and skeletal muscle cells (**B**). Downward arrows indicate decreased levels of parameters. IR, ischemia reperfusion; MPO, myeloperoxidase; mPTP, mitochondrial permeability transition pore; ROS, reactive oxygen species.

It has been found that ROS accumulated rapidly at the onset of ischemia in the heart of rats [34]. The potential sources of ROS during ischemia are linked with impairment of the mitochondrial respiratory chain and oxidation of ferrous heme (Fe^2+^) in the oxymyoglobin (Mb) complex [6,34]. Specifically, Fe^2+^ is converted into ferric heme (Fe^3+^) during ischemia, which is coupled with the formation of O_2_^•−^ [34]. When the following reperfusion is initiated, a burst of ROS occurs via the aforementioned reaction (1) catalyzed by XO [30]. Additionally, neutrophils are recruited and activated following IR, which release toxic oxidants to myocardium (Table 1) [35]. The significant cell death observed during cardiac IR injury is mainly associated with the oxidative stress-mediated opening of mPTP [36,37]. Specifically, the activation of mPTP results in the hydrolysis of mitochondrial ATP as well as the dysfunction of ATP-driven Ca^2+^ pumps, which ultimately leads to cytosolic Ca^2+^ overload and cell necrosis [36]. Thus, the inhibition of mPTP was found to effectively prevent Ca^2+^ overload in cardiac myocytes [36]. Furthermore, the opening of mPTP initiates apoptotic cascades via the release of cytochrome *c* from mitochondria [37]. While ischemic necrosis does cause substantial cell death in IR, the reperfusion period can lead to an additional 25–40% cardiac cell death. This is due to the altered metabolism of ischemia-damaged mitochondria which can lead to excessive ROS production, dysregulation of calcium, and swelling and disruption of the mitochondria caused by mPTP opening (Figure 1; Table 1) [38]. 

Interestingly, subsarcolemmal mitochondria (SSM) are more prone to ischemic injury than interfibrillar mitochondria (IFM) in cardiac muscle [38]. Specifically, SSM are more susceptible to cytochrome *c* release mediated by Ca^2+^ overload due to a lower capacity for calcium accumulation compared to IFM [38]. Ischemia damages mitochondria in a progressive fashion, beginning with complex I, which causes excessive ROS production. It also affects complex V and the adenine nucleotide transporter [38]. SSM in vitro display a greater tendency to release cytochrome *c* compared to IFM due to elevated external Ca^2+^ concentrations, making SSM more likely to be affected by programmed cardiomyocyte death. Additionally, cardiolipin, a key phospholipid component of inner mitochondrial membranes, has been shown to be markedly reduced during ischemia [38]. Since cardiolipin provides binding sites on the inner membrane for cytochrome *c*, loss of cardiolipin may lead to increased release of cytochrome *c* into the mitochondrial intermembrane space, which renders the cells sensitive to apoptotic signals [38,39]. In addition, it was reported that ROS-mediated cardiolipin peroxidation significantly compromises complex III activity within the respiratory chain during IR. The disruption of complex III can further increase ROS formation, which eventually contributes to heart failure [40]. Specifically, Ca^2+^ leakage from the ryanodine receptor2 (RyR2) has been reported to cause mitochondrial dysfunction and ROS production. The increased ROS creates a feedback loop by oxidizing RyR2 receptors, thus increasing the severity of the Ca^2+^ leak [41].

### 3.2. ROS-Mediated IR Injury in Skeletal Muscle

During ischemia, diminishing ATP levels are closely linked with muscle necrosis, although the magnitude of ATP reduction in skeletal muscle is smaller than that in cardiac muscle (Table 1) [42,43]. ROS and neutrophils have been suggested as the major mediators responsible for IR-induced injury in skeletal muscles. Ischemia induces ROS production via the activation of XO in endothelial cells in skeletal muscle. This ROS generation can be exacerbated during the reperfusion stage [1]. Excessive ROS accumulation may impair cell membranes via lipid peroxidation. The byproducts of cell injury activate lipoxygenase pathways, which in turn triggers neutrophil activation [1]. During reperfusion, activated leukocytes release large amounts of ROS and cytotoxic enzymes, resulting in the leakage of plasmatic proteins [44]. These events cause substantial changes in transcapillary permeability, which leads to increased interstitial fluid pressure and capillary compression. As a result, myocytes undergo necrosis due to both the deficiency of metabolic nutrients and the direct attack by leukocytes [1,44]. In addition, myeloperoxidase (MPO) released from the activated neutrophils has been implicated in IR injury due to its ability to convert H_2_O_2_ and chloride ions into highly toxic hypochlorous acid (HOCl) and other oxidants (Figure 1) [2,45]. These products cause significant damage in cell membranes. Moreover, HOCl may react with H_2_O_2_ to produce single oxygen (^1^O_2_), which exacerbates the peroxidation of membrane lipids and myocyte death (Table 1) [1].

## 4. ROS-Mediated Protection against IR Injury

Given that oxidative stress is a major contributor to IR injury, numerous studies have been performed to investigate the effectiveness of antioxidants in alleviating IR-induced muscle damage [47,48,49]. For example, mitoQ, a selective mitochondrial ROS inhibitor, has been shown to dramatically attenuate heart dysfunction and mitochondrial damage after IR in rats [47]. In addition, a recent study has shown that saffron extract, a stemless herb that exhibits antioxidant capacities, decreased IR-related oxidative stress in rat skeletal muscles when administrated one hour before reperfusion [49]. Despite these positive outcomes from antioxidant application, some contradictory results have also been reported [6]. For instance, a clinical trial has shown that intravenous administration of human SOD did not provide any ventricular protection for patients who received percutaneous transluminal coronary angioplasty following reperfusion [50]. Thus, more studies are necessary to investigate the effectiveness of antioxidants in protecting striated muscles during IR.

Despite the significant adverse effects of ROS overproduction on striated muscle function, increasing evidence has suggested that a small amount of ROS release is essential for cardioprotection [51]. Matsushima et al. demonstrated that NOX-2 or NOX-4 induces low levels of ROS formation, activating hypoxia-inducible factor (HIF)-1α during IR. Hypoxia-inducible factor-1α (HIF-1α) mediates hypoxia-related adaptations via the upregulation of glycolytic genes, maintaining ATP supply during ischemia [51]. Therefore, the inhibition of NOX-2 or NOX-4-derived ROS production is unfavorable, since it may exacerbate IR-induced myocardium injury [51]. Furthermore, beneficial roles of ROS have also been intensively studied in the protective mechanisms of IPC [6,52].

### 4.1. Redox Mechanisms of IPC in Cardiac Muscle

IPC, which consists of a few cycles of muscle exposure to sublethal levels of IR, has been shown to provide effective protection for both cardiac and skeletal muscles during IR [6,52]. Preconditioned myocardium exhibits attenuated oxidative stress during prolonged IR exposure [6]. The underlying mechanisms for this protection in cardiac muscle involves the opening of mitochondrial ATP-sensitive K^+^ (mitoK_ATP_) channels and a small amount of ROS generation, as either the inhibition of mitoK_ATP_ or ROS production can nullify the protection offered by IPC [53]. Specifically, the activation of mitoK_ATP_ may trigger mitochondrial ROS formation, which in turn inhibits mPTP opening via protein kinase C (PKC) activation and prevents cell death [54]. PKC is an important signaling molecule in the IPC cycle that allows for the protection of mitochondria. In the initial phase of IPC, opioid, bradykinin, and adenosine receptors are normally activated to trigger PKC and sensitize the A2b adenosine receptor (A2bAR) [55]. Sensitized A2bAR triggers the activation of a survival pathway involving PI3 kinase, Akt, and ERK, which is responsible for mPTP inhibition [55,56]. Activation of mitoK_ATP_ has been shown to prevent mitochondrial matrix contraction, which could induce beneficial effects by improving ATP synthesis [6].

### 4.2. Redox Mechanisms of IPC in Skeletal Muscle

The mitoK_ATP_-dependent pathways have also been indicated to play a central role in IPC protection in skeletal muscles [53,57]. Animal studies have shown that the application of mitoK_ATP_ openers such as diazoxide significantly improved ischemic tolerance of skeletal muscles [58]. These protective effects have been attributed to ATP sparing effects and neutrophil inhibition. Specifically, IPC is associated with reduced ATP consumption during ischemia and lower MPO activities following reperfusion in skeletal muscles [8]. It was speculated that the opening of mitoK_ATP_ channels caused a reduction in mitochondrial ATPase activity and ATP hydrolysis rate, which spare ATP during prolonged ischemia. Additionally, expression levels of endogenous antioxidant enzymes can be elevated after IPC treatment [59]. Direct IPC, defined as IPC performed at a target organ, increased the expression of SOD2 and catalase genes while remote IPC, performed at a remote site, increased the expression of GPx, SOD2, and catalase genes. Accordingly, this enhancement of antioxidant expression can elicit a potential protection exerted on skeletal myocytes against oxidative stress [59].

## 5. IR-Induced Injury in Aging and Diabetes

IR-associated mortality is significantly higher in the elderly as compared to that in young adults [60]. Aging heart is characterized by compromised cardiac function, which is partially attributed to the increased vulnerability to oxidative stress [61]. Higher levels of protein oxidation were observed in senescent than in young heart of rats [62]. It was suggested that the ischemic tolerance of heart started to decrease from the age of 12 months in C57BL6 mice [63]. Apart from aging, the development of IR injury can be potentially affected by pathologic conditions such as hypertension, diabetes, hyperlipidemia, and insulin resistance. Among these diseases, diabetes is one of the most important factors that can greatly influence the severity of IR-mediated injury [60]. According to the American Diabetes Association, there are about 30.3 million cases of diabetes in 2015 in the USA, which indicates that 9.4% of the US population had diabetes [64]. In 2015, diabetes contributed to 252,806 deaths, and remained the seventh leading cause of death [64]. In fact, more than 50% of the diabetes-related deaths are caused by ischemic heart disease [60]. Treatment of diabetic patients with ischemic heart disease is particularly challenging due to the complex pathophysiology and the poor prognosis of comorbidities [60]. Therefore, it is imperative that researchers and physicians work closely to develop individualized treatment strategies against IR injuries for these patients.

### 5.1. Aging Effects on IR Injury

Aging and diabetes are two major risk factors for coronary heart disease [65,66]. IR-induced morbidity and mortality could be more significant in the elderly and diabetes-affected populations [9,10]. Evidence has shown that aging can increase cardiac susceptibility to IR injury [10,67,68], and cause more severe myocardial dysfunction [68]. The exacerbated damaging effects have been attributed to a decline in thioredoxin (Trx) activity and augmented oxidative stress observed in an elderly heart [67,68]. As compared with younger patients, IR resulted in significantly higher levels of plasma malondialdehyde (a marker for lipid oxidation) and lower glutathione (GSH) concentrations in elderly patients [68,69]. This is potentially due to the compromised antioxidant activities in an aging heart. For example, the cytosolic CuZn SOD and GPx levels were found to be much lower in the heart of old mice (26 and 31 months old) in comparison to a young mouse (4 months old) [70]. Furthermore, it was reported that Trx displayed lower activities in aging hearts as compared to young hearts and that this difference can be exacerbated after IR [67]. Since Trx is a critical cytoprotective molecule that mediates the action of other antioxidants, a decline in Trx activity may precipitate ROS formation, thereby increasing susceptibility of the elderly to IR injury [2]. Moreover, it was found that the aging heart showed no response to IPC or pharmaceutical preconditioning (e.g., PKC analog or mitoK_ATP_-channel opener). The protective effects were also attenuated in middle-aged hearts compared to young hearts. These results indicated a decreased sensitivity with aging in response to preconditioning stimuli [71]. Therefore, alternative therapies are needed to protect the aging population against IR injuries.

Aging is able to impact the physiology of skeletal muscle in aspects of regenerative capability, repair process, inflammatory reaction, and oxidative stress [72,73,74]. These age-related alterations can potentially affect the outcomes of IR injury in skeletal muscle. For example, it was found that macrophage infiltration was significantly reduced in the skeletal muscle of the elderly as compared to young men after exercise-induced injury [73]. Since activated leukocytes account for an important source for ROS during reperfusion, it is likely that the ROS production from activated macrophages was reduced in the elderly compared to young men following IR [44]. However, low levels of inflammatory cells may also contribute to a slower regenerative process of muscle in the elderly [44]. Contradictory results were reported by Ghaly et al., who observed greater infiltration of macrophages in skeletal muscle samples of the old mice in comparison to young mice after contusion injury [75]. In addition, aged muscle is characterized by higher levels of oxidative stress either under normal condition or after prolonged exercise [76]. Therefore, it remains to be elucidated whether IR-induced ROS formation is worsened in the elderly as compared to young individuals. Further studies are needed to explore the potential effects of aging on IR injury in skeletal muscle.

### 5.2. Effects of Diabetes on IR Injury

Mixed results have been produced in terms of determining diabetes relating to IR injury [9,77]. For example, a study by Engbersen et al. suggested that patients with type I diabetes demonstrated higher resistance to IR-induced injury than non-diabetic controls in the forearm [77]. In their IR protocol, the blood circulation of nondominant forearm was occluded by the inflation of an arm cuff for 10 min with simultaneous isometric hand gripping exercise to induce ischemia followed by reperfusion [77]. Radiolabeled annexin V5 was intravenously injected as an indicator for muscle damage. The extent of IR injury was determined four hours after injection by targeting annexin V5 in the arm using a gamma camera [77]. Diabetic patients showed markedly reduced levels of annexin V5 compared to healthy volunteers following IR, indicating a higher tolerance to IR injury in diabetic patients [77]. The study also examined the protective effects of IPC in diabetic patients compared to healthy individuals. Interestingly, it was found that although IPC exerted prominent protection against IR injury in healthy volunteers, it did not reduce the IR injury in diabetic patients. These results suggest that the protective effects conferred by IPC on skeletal muscle can be abolished due to type I diabetic conditions [77].

However, opposing evidence showed that IR induced a larger infarct size in the hearts of rats with type I diabetes as compared to heathy rats. In particular, more severe oxidative stress and less NO bioactivity was observed in diabetic hearts, which may account for the muscle sensitivity to IR injury [9]. The main sources of free radicals in the diabetic heart are NOX, mitochondria, and NOS [78]. Elevated NOX activity, lipid peroxidation, and O_2_^•−^ production are evident in type II diabetic mice, with potential oxidative damage exacerbated by decreased enzymatic and non-enzymatic antioxidant activity [78]. Furthermore, research has shown that the efficacy of IR therapies (e.g., IPC and postconditioning) was compromised by diabetes mellitus [77]. However, it was reported that the activation of peroxisome proliferator-activated receptor may be effective in protecting the type-II diabetic rat heart against IR injury [79]. Antioxidant supplementation has been shown to decrease levels of plasma markers of lipid peroxidation [78]. Since current results regarding the effects of diabetes on IR injury are inconsistent, further investigation is needed to understand the interplay between the pathology of diabetes and IR-mediated muscle injury. 

## 6. Recent Advances in Preventing IR Injury

Apart from IPC, postconditioning has been recently proposed as an alternative strategy to protect against IR injury in both cardiac and skeletal muscles [80,81]. Postconditioning follows similar protocols as IPC by alternately exposing the muscles to short periods of ischemia and reperfusion; however, the treatment is given at the beginning of reperfusion instead of before ischemia [80]. Compared to IPC, postconditioning is more clinically relevant because it is usually impossible to provide IPC before the onset of ischemia and prior to unpredictable myocardial infarction and extremity injury. Similar protective pathways have been identified underlying postconditioning in the heart, which involves the activation of PKC and ROS formation [6,54]. Briefly, postconditioning initiates moderate ROS generation at the onset of reperfusion, which can trigger the opening of the mitoK_ATP_ channel. The activation of mitoK_ATP_ leads to secondary ROS formation from mitochondria, which in turn inhibits mPTP opening to prevent cell death [6,54]. In skeletal muscles, postconditioning was found to improve antioxidant activities and decrease Bcl-2/Bax expression [82]. Since both Bcl-2 and Bax are key mediators in regulating apoptosis, postconditioning potentially protects against IR-induced skeletal muscle injury via inhibition of cell apoptosis [82].

Furthermore, pharmacological interventions that target the pre- or postconditioning pathways hold great potential in alleviating IR injury [83]. For instance, cyclosporin, an mPTP blocker administrated at the time of reperfusion, has been reported to reduce IR-induced myocardial infarction in patients after reperfusion [84]. Additionally, recent work by Arslan et al. found that the key features of IR injury, including augmented oxidative stress and loss of ATP/NADH, are associated with proteomic deficiencies of the heart, which can be mitigated by exosome secretion from mesenchymal stem cells [85]. Therefore, they examined the protective effectiveness of exosomes on the mouse heart following IR. It was found that exosomes successfully attenuated myocardial infarction and oxidative stress following IR. These findings suggest a promising therapy of using exosomes to protect against IR-related striated muscle injuries [85].

In addition, mitochondria-targeted antioxidants appear to be more therapeutically favorable than cellular ROS scavengers in animal studies [86]. For example, SS31 is an antioxidant peptide that specifically targets mitochondria to scavenge ROS production and inhibit mPTP opening [87]. The effects of SS31 in the rat model are more noticeable in older subjects, suggesting that pathological ROS formation in aging mitochondria can be better targeted by this peptide [86]. Targeting defective mitochondria provides protective measures by reducing excess ROS without affecting physiological levels of ROS. The mitochondria of cardiomyocytes are commonly fragmented in heart failure and other pathological conditions that cause ROS production [86]. The inhibition of Drp1 (mitochondria fission promoter) via P110 was found to successfully reduce pathological levels of mitochondrial fragmentation during IR injury in rat cardiomyocytes [86,88,89]. The study found that mitochondrial fissions were markedly reduced in cardiomyocytes during IR injury. Importantly, during normal conditions, P110 had neither adverse effects on ROS levels nor any induction of cell death [86]. In recent studies, low-level laser therapy (LLLT) has been proposed as a novel treatment that can significantly reduce the infarct size caused by IR by stimulating muscle cell regeneration [90]. It was found that LLLT applied to the infarcted area of heart can markedly attenuate the scar tissue formation in animal models. Studies on skeletal muscle showed similar results where LLLT substantially reduced the degeneration of rat skeletal muscle following IR [91]. The protective effects of LLLT have been attributed to its ability to promote angiogenesis, the synthesis of heat shock proteins and inducible nitric oxide (NO) synthase. Further, LLLT has stimulatory effects on cardiac stem cells and MSCs proliferation, which underlies its protective mechanisms of limiting muscle infarct size [90]. The common protective strategies against IR injury are summarized in Table 2.

## 7. Conclusions and Future Perspectives

IR-induced injury has become a serious clinical concern that causes significant morbidity and mortality, especially in the aging and diabetic populations. ROS-mediated oxidative stress has been suggested to play a major role in the pathogenesis of IR [6]. Accordingly, protective strategies that target the ROS-centered pathways have been proposed to prevent IR injury, such as IPC, postconditioning, and pharmacological interventions [48,80,81]. Previous studies have produced different results regarding the protective effects of these treatments on cardiac and skeletal muscle; however, great advances have been made in understanding the interplay between ROS, antioxidants, and IR-mediated injury. Future research is warranted to standardize the treatment modalities of pre- or postconditioning as well as to develop reliable pharmacological agents to enhance the protective efficacy of existing therapies.

## Figures and Tables

**Table 1:** Characteristics of cardiac and skeletal muscle injury and major sources of ROS during ischemia and reperfusion.

		Cardiac Muscle	Skeletal Muscle
**Ischemia**	Characteristics	ATP depletion [43] Cell necrosis [46] Excessive ROS generation [6]	ATP depletion [30] Cell necrosis [42] Excessive ROS generation [31]
	Major sources of ROS	Impaired mitochondrial respiratory chain [6] Oxymyoglobin [34] Activation of XO [2]	Activation of XO [1]
**Reperfusion**	Characteristics	Excessive ROS generation [32] mPTP opening [36,37] Ca^2+^ overload [36] Cytochrome *c* release [37] Cell swelling [38] Cell necrosis and apoptosis [36,38]	Excessive ROS generation [30] MPO activation [1] Lipid peroxidation [1] Leukocyte activation [1] Capillary compression [1] Cell necrosis [7,8]
	Major Sources of ROS	XO [6] Neutrophils [6]	XO [1] Neutrophils [1] MPO [45]

MPO, myeloperoxidase; mPTP, mitochondrial permeability transition pore; ROS, reactive oxygen species; XO, xanthine oxidase.

**Table 2:** Protective strategies against IR-induced injury in cardiac and skeletal muscle.

Cardiac Muscle			Skeletal Muscle		
Protective Strategies	Protective Effects	Animal Models	Protective Strategies	Protective Effects	Animal Models
IPC	Reduced infract size	Dogs [92]	IPC	Reduced infarct size Lowered energy metabolism	Pigs; latissimus dorsi and gracilis muscles [52]
		Pigs [93]			
	Attenuated arrhythmias	Dogs [94]			
		Rats [95]			
	Improved recovery of cardiac function Improved SR Ca^2+^ release Improved Ca^2+^-pump protein contents Improved Ca^2+^/calmodulin-dependent protein kinase phosphorylation	Rats [96]		Decreased MPO in muscle Reduced NO metabolites and TNF-α levels in blood	Rats; gastrocnemius muscle [97]
Remote IPC	Reduced cardiac troponin I Attenuated chest discomfort during PCI Reduced adverse cardiac and cerebral event rate after PCI	Human [98]	Remote IPC	Reduced infarct size	Pigs; latissimus dorsi, gracilis, and rectus abdominis muscles [8]
Ischemic Post-conditioning	Reduced infarct size	Dogs [80] Rats [99] Rats [100]	Ischemic Post-conditioning	Reduced muscle MPO activity Decreased mitochondrial free Ca^2+^ concentration Increased muscle ATP content	Pigs; latissimus dorsi muscle [81]
	Reduced lipid peroxidation and superoxide generation	Dogs [80] Rats [99]		Decreased lipid peroxidation Reduced creatine kinase activities Improved antioxidant defense Reduced muscle cell apoptosis	Rabbits; limbs [82]
	Reduced tissue edema Reduced polymorphonuclear neutrophil accumulation Improved endothelial function	Dogs [80]			
	Reduced creatine kinase activity	Rats [99]			
Cyclosporine (an mPTP inhibitor)	Reduced infarct size Reduced creatine kinase release	Human [84]	Cyclosporin A (an mPTP inhibitor)	Reduced muscle MPO activity Decreased mitochondrial free Ca^2+^ concentration Increased muscle ATP content	Pigs; latissimus dorsi muscle [81]
Melatonin	Attenuated arrhythmias	Rats [101]	Melatonin	Reduced O_2_^•−^ formation in arterial walls Improved myocyte viability Reduced microvascular endothelial dysfunction	Rats; cremaster muscle [102]
	Reduced infarct size Prevented mitochondrial cytochrome *c* release Inhibited mitochondrial mPTP opening	Rats [103]		Reduced oxidative stress Reduced muscle damage	Rats; hindlimb [104]
Low-Level Laser Therapy	Reduced infarct size Reduced ventricular dilation Increased cardiac stem cell density in infarct area	Rats [90]	Low-Level Laser Therapy	Reduced muscle degeneration	Rats; gastrocnemius muscle [91]
Exosomes	Reduced infarct size Improved contractile function Decreased oxidative stress Reduced local and systemic inflammation	Mice [85]	Remote Post-conditioning	Reduced MPO activity Decreased tissue necrosis	Mice; hindlimb [105]
SS-31 (a mitochondria-targeted peptide)	Reduced infarct size	Pigs [106]	Adenosine Treatment	Decreased MPO in muscle Reduced NO metabolites and TNF-α levels in blood	Rats; gastrocnemius muscle [97]
		Rats [107]		Higher phosphocreatine and ATP levels during ischemia Lowered dephosphorylated metabolites and lactate during ischemia	Pigs; latissimus dorsi muscle flap [108]
	Reduced lipid peroxidation Reduced arrhythmia	Rats [107]	*S*-Nitroso-*N*-Acetylcysteine	Improved muscle contractile function	Rats; EDL [25]

EDL, extensor digitorum longus; IPC, ischemic preconditioning; MPO, myeloperoxidase; mPTP, mitochondrial permeability transition pore; PARP, Poly(ADP-ribose) Polymerase; PCI, percutaneous coronary intervention; TNF-α, tumor necrosis factor-alpha.

## Abbreviations

AMPK	AMP-activated protein kinase
ATII	Angiotensin II
A2bAR	A2b adenosine receptor
EDL	Extensor digitorum longus
ERK	Extracellular signal-regulated kinases
GPx	Glutathione peroxidase
HIF-1α	Hypoxia-inducible factor-1α
IFM	Interfibrillar mitochondria
IPC	Ischemic preconditioning
IR	Ischemia reperfusion
JNK	Jun amino-terminal kinases
MAPKs	Mitogen-activated protein kinases
mitoK_ATP_	Mitochondrial ATP-sensitive K^+^
MPO	Myeloperoxidase
mPTP	Mitochondrial permeability transition pore
NAC	N-acetylcysteine
NOX	NADPH oxidase
PARP	Poly(ADP-ribose) Polymerase
PCI	Percutaneous coronary intervention
PKC	Protein kinase C
ROS	Reactive oxygen species
RyR	Ryanodine receptor
SERCA2	Sarcoplasmic reticulum Ca^2+^ ATPase 2
SOD	Superoxide dismutase
SSM	Subsarcolemmal mitochondria
TNF-α	Tumor necrosis factor-alpha
Trx	Thioredoxin
